# Evidence-based medicine among internal medicine residents in a community hospital program using smart phones

**DOI:** 10.1186/1472-6947-7-5

**Published:** 2007-02-21

**Authors:** Sergio A León, Paul Fontelo, Linda Green, Michael Ackerman, Fang Liu

**Affiliations:** 1 Office of High Performance Computing and Communications, 8600 Rockville Pike, National Library of Medicine, National Institute of Health, Bethesda, MD 20894, USA; 2 Internal Medicine Residency Program, Prince Georges Hospital, 3001 Hospital Drive, Cheverly, MD 20785, USA

## Abstract

**Background:**

This study implemented and evaluated a point-of-care, wireless Internet access using smart phones for information retrieval during daily clinical rounds and academic activities of internal medicine residents in a community hospital. We did the project to assess the feasibility of using smart phones as an alternative to reach online medical resources because we were unable to find previous studies of this type. In addition, we wanted to learn what Web-based information resources internal medicine residents were using and whether providing bedside, real-time access to medical information would be perceived useful for patient care and academic activities.

**Methods:**

We equipped the medical teams in the hospital wards with smart phones (mobile phone/PDA hybrid devices) to provide immediate access to evidence-based resources developed at the National Library of Medicine as well as to other medical Websites. The emphasis of this project was to measure the convenience and feasibility of real-time access to current medical literature using smart phones.

**Results:**

The smart phones provided real-time mobile access to medical literature during daily rounds and clinical activities in the hospital. Physicians found these devices easy to use. A post-study survey showed that the information retrieved was perceived to be useful for patient care and academic activities.

**Conclusion:**

In community hospitals and ambulatory clinics without wireless networks where the majority of physicians work, real-time access to current medical literature may be achieved through smart phones. Immediate availability of reliable and updated information obtained from authoritative sources on the Web makes evidence-based practice in a community hospital a reality.

## Background

Meeting the information needs of busy physicians at the point of care is an important challenge in medicine. Finding the best evidence to answer clinical questions is one of the basic steps in evidence-based medicine (EBM) practice [1]. To be most effective, the practice of EBM must occur in real-time at the point of patient care because physicians almost never seek answers to clinical questions after the clinical session ends [2,3]. Furthermore, medical residents answering patient-specific questions reported improvement in knowledge and changes in patient care decisions [4].

The Internet has had a major impact on physicians' practice by improving their access to medical information resources. Clinicians frequently use online evidence primarily to support clinical decisions related to direct patient care [5]. Physicians' use of the Internet and PDAs is growing [6-8], with 60% to 70% of medical students and residents using PDAs for educational purposes and patient care in 2006 [9]. However, in clinical practice there may be limited availability of desktop computers, Internet access or wireless networks. Wireless networks that allow ubiquitous access to online information through portable mobile devices are now common in major academic medical centers, six percent of the US hospitals in 2000 [10]. However, they may not be available in many community hospitals and ambulatory clinics where the majority of physicians practice. The American Hospital Association reported that community hospitals represented 85% of the total number of hospitals in the U.S in 2004 [11].

Smart phones – new hybrid devices that combine the communication capabilities of mobile phones with easy and fast access to the Web and computing features of PDAs – may be an effective way to provide real-time access to online medical knowledge resources at the bedside. We were unable to find studies on this type of utilization of smart phones, so we implemented a wireless Internet access project to evaluate the feasibility of using smart phones as an alternative to reach online medical resources for information retrieval during clinical and academic activities in a community hospital. In addition, we wanted to learn what Web-based information resources internal medicine residents were using before the study, if there were any differences in the group related to the level of training, and whether providing bedside, real-time access to medical information would be perceived useful for patient care and academic activities.

## Methods

### Settings

The study was performed at Prince George's County Hospital, a 290-bed, acute care teaching hospital and regional referral center located in Cheverly, Maryland, from August 2005 to February 2006. The hospital sponsors an internal medicine residency program with 42 residents. There are four medical teams in the wards; each one consists of a senior resident, two first-year residents (Interns), and two medical students under the supervision of an attending physician. All team members are changed every month to allow rotation of residents in the hospital wards. The general medical teaching service habitually admits ten to twelve patients in a twenty-four hour period. Medical residents and staff attendings have a subscription to MDConsult provided by the hospital. On average, there are three to four desktops available for physician's use at each nurse station. They are heavily used for Internet access, laboratory results, radiology reports, and patient records. There is no 802.11 wireless network in the hospital.

The study met the requirements of the Research Committee of the Prince George's Hospital Center; temporary approval was granted on July 14, 2005 and final approval on September 13, 2005. All participants signed informed consent. The study had two main components, an initial cross-sectional survey to assess the patterns of Internet and handhelds usage by residents and attendings of the internal medicine department, followed by a prospective interventional cohort study to address the feasibility of using smart phones to access online medical resources during daily clinical activities.

### Pre-study Internet and handhelds use survey

The survey was conducted prior to the smart phones experience to assess the level of knowledge on medical Web resources among residents and staff attendings of the department, as well as to evaluate the patterns of usage of the Internet and PDAs among the group and if there were any differences related to the level of training and medical practice [see Additional file 1]. The survey was distributed on paper and filled during the department meetings and academic activities to allow all the house staff to complete. No incentives were offered to the respondents.

### Smart phones cohort study

Special lectures were given on medical informatics, mobile computing and EBM practice. These lectures as well as training sessions and group workshops on the use of the smart phones and Medline searching tools available at the U.S. National Library of Medicine (NLM) were carried out before and during the period of the study as part of the monthly conference schedule of the Department of Internal Medicine. Additional one to one training was provided by the resident in charge of the project in order to instruct other residents, students and attendings on the use of the resources.

The NLM lent three Palm Treo650^® ^smart phones for the medical teams. Phone service and unlimited wireless Internet access through the T-Mobile network was obtained by the hospital at a monthly cost of US$120. The phones were assigned on a monthly basis to medical teams rotating in the wards. They were available for the teams' use from 8 am to 4 pm, Monday to Friday and locked at the Internal Medicine Department's office during nights and weekends. The devices were used by team members under the attending physician's supervision to search for answers to clinical questions when the need arose during rounds, morning reports, noon conferences and other academic activities.

**Figure 1:**
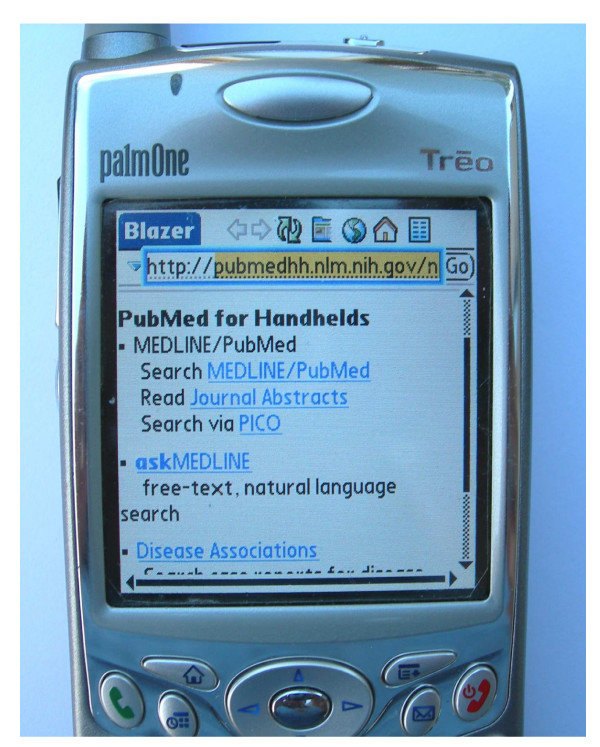
Index page of "PubMed for Handhelds" on the Palm Treo650 screen.

Evidence-based medicine resources developed at the NLM were used as gateways for accessing published articles. The smart phones' index page was "PubMed for Handhelds"  [Figure 1]. This handheld-friendly Web page provided links to: MEDLINE, *ask*Medline, Disease Associations (DA) and Patient Intervention Comparison Outcome (PICO). An access log was created on the NLM server through IP number identification of the mobile phones to observe smart phones utilization. These IP numbers were determined by testing and confirmed with the wireless service provider. Access to Websites outside of the NLM was not monitored.

All the interactions were Web-based and no special software was required. The teams had no restriction to access other on-line medical resources they considered important during their searches. We obtained feedback from users after their one-month rotation by a final questionnaire on usage and satisfaction with the smart phones, efficacy of real-time access to references, perception of the value of the medical information derived from Internet and self-reported global impact in the decision-making process [see Additional file 2]. In the study, we considered a search as effective when the information retrieved using smart phones resolved the specific clinical question and could be used for the team's discussion at the bedside regarding the diagnosis or management of a patient. In academic activities, the medical information was considered positive if it contributed new knowledge, brought up new medical advances or new thoughts that generated structured analysis.

## Results

### Pre-study Internet use survey

The initial survey was given to 63 physicians and completed by 60; five faculty attendings and all 55 residents rotating in the internal medicine program during July and August 2005, the first two months of the training year. They were divided in two groups based on the specialty level of training and practice, twenty-four were new first year residents (Interns group) and 36 were senior residents or faculty attendings (PostPGY1 group). Although not all the questions were answered, the entire group of participants (100%) reported habitual use of the Internet, accessing the Web usually from desktop computers [Table 1]. Access from the hospital was more frequent in the PostPGY1 group (55% of the times) while it was more frequent from home in the Interns group (68%). Fifty-seven of the 60 physicians interviewed (95%) used the Internet on a daily basis [Table 2]. Thirty-one doctors (52%) reported a daily usage of less than five times per day whereas five physicians (8%) used the Internet more than 10 times a day at that time. The reported time spent on the Internet was between one to two hours a day for the majority of the group (64%) [Table 3]. Only three of the physicians in the Interns group were not using the Internet on a daily basis – their reported usage was three to four times per week.

We found differences between the two groups in the usage of the Internet [Table 4]. The Interns group spent more time on personal information activities, including e-mail (43%) whereas the PostPGY1 group spent more time on patient clinical information (30%). The second most common use by both groups was searching for general medical or scientific information. The groups used fairly similar Websites to search for general scientific information but their pattern of use was slightly different. The five sites most commonly mentioned were UpToDate, Google, eMedicine, PubMed and MDConsult [Table 5]. If the questions related to specific issues concerning patient management, the preferred Websites were UpToDate, PubMed, MDConsult, Google and New England Journal of Medicine (NEJM) [Table 6]. When asked about the resources they accessed for evidence-based medicine, both groups mentioned similar choices, the four most common were: UpToDate, NEJM, PubMed and MDConsult [Table 7] When asked about their interest in getting more education and training in the use of Internet for medical applications, 83% of the PostPGY1 and 95% of the Interns were interested on medical knowledge resources, tools for clinical practice and EBM resources training [Table 8].

### Pre-study handhelds use survey

Not all questions were answered by the group. Twenty of the 60 physicians interviewed (33%) owned a PDA at the time of the survey, sixteen in the PostPGY1 group and four in the Interns group. Ten of them had PDAs for one to three years and nine for less than a year [Table 9]. Seven of the handhelds were Palm devices and the others were Pocket-PC, including Dell (6), Sony (3), and HP (1), or were unidentified. Although 13 of the PDAs were wireless-enabled they were not being used for Internet access to obtain real-time medical information. None of the physicians had previous formal training on the use of these devices and 75% of them reported self learning from manuals or learned from peers. Fifteen physicians who had PDAs (75%) reported using it daily [Table 9]. They used the handhelds on average seven times a day (range: 1–20). Their main usage was for patient care resources, including drug information programs (pharmacopoeias), medical references and clinical tools [Table 10]. Most of the 40 physicians who did not own a handheld at the time of the survey were planning to buy one in the near future – twelve in the PostPGY1 group (60%) and 14 in the Interns group (70%).

### Evaluation of smart phones usage

From the group of 55 residents that answered the pre-study survey, thirty-one used the smart phones during the seven-month trial period – thirteen in the PostPGY1 group and 18 in the Interns group. All of them filled the post-study evaluation and reported that this was their first experience with real-time mobile Internet access for clinical activities. During the time of the study, twenty-five physicians (80%) reported accessing the Web from the smart phones between 1 to 5 times a day and four of them between 5 to 10 times, only two physicians reported accessing the Internet more than 10 times a day.

**Figure 2:**
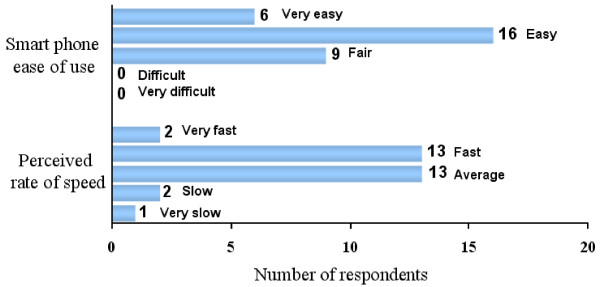
**Ease of use and perceived rate of speed**. Perceived usability of the smart phones and rate of speed (n = 31)

We monitored and measured the usage of NLM resources but the participants also used the smart phones to access other Websites for their searches. The most common reported were UpToDate, eMedicine, MDConsult, New England Journal of Medicine, Google and Medscape. The smart phones were found "very easy" or "easy" to use by twenty two of the physicians (71%), whereas nine of them considered their usage as "fair". None of them considered that their use was difficult [Figure 2]. Thirteen physicians rated the speed of the wireless connection as "fast" and a similar number considered that it was "average". On the other hand, only three of the participants (10%) rated the speed of the Internet connection as slow or very slow [Figure 2]. Residents reported a slow transmission from some Websites that were not handheld-friendly, making their access a time-consuming process.

Eighteen of the 31 physicians (58%) reported that they "frequently" found the information they were looking for, ten of them found it "sometimes" (32%), and three doctors in the Interns group reported that they "always" found the proper information during their searches [Table 11]. Sixteen physicians (52%) considered that the information obtained at real-time "frequently" had an impact in the diagnostic or management process of patients, whereas for 13 participants it happened "sometimes" (42%) and "rarely" or "never" for only two physicians from the Interns group [Table 12]. Twenty-nine of the participants said that they were "satisfied" or "very satisfied" with the experience (94%), and the same number stated that they were "likely" or "very likely" to use this technology in the future. Moreover, twenty-three of them (74%) were planning to buy a PDA with wireless Internet access for personal use in the future. All the participants would recommend the daily use of these devices to colleagues in their practices.

The final evaluation included a section for free comments. We did not measure the actual time spent doing the searches during the rounds, but the work of the team was not affected by the use of the smart phones because usually only one of the team members was in charge of the literature search while the others continued assessing the patient. In general, house staff commented that they saved time using the phones because of the "immediate availability of information for discussion of patients' medical problems". Physicians considered the small size and mobility as main advantages of these devices. This availability and easy access to medical, scientific information saved time during the daily activities "when it is difficult to find a desktop available" or "from any place in the hospital". The smart phones were easy to carry and allowed fast and ubiquitous access to the Internet. They also commented on better results "when proper questions were made" and there was not "impatience for developing analysis". Residents and faculty participating in this study reported that the information retrieved from the Internet was used not only for discussions about specific cases but also to review topics with attendings, update individual knowledge and prepare academic activities such as morning reports, journal clubs and noon conferences.

The lack of familiarity with smart phones and the small keyboard and screen were reported as negative factors for usability. Other barriers or disadvantages mentioned were: "cost of the equipment", "phone company charges", "large amount of information needed every day", and "physician's impatience". There were no reports of interference of the cellular phones with medical devices during the study period.

### NLM server logs analysis

**Figure 3:**
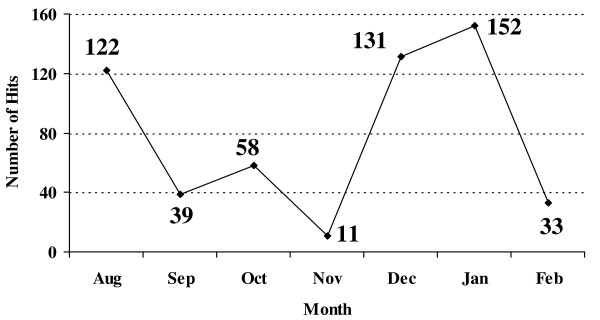
**Monthly Access to NLM Server**. Number of hits to NLM server by month from August 2005 to February 2006

**Figure 4:**
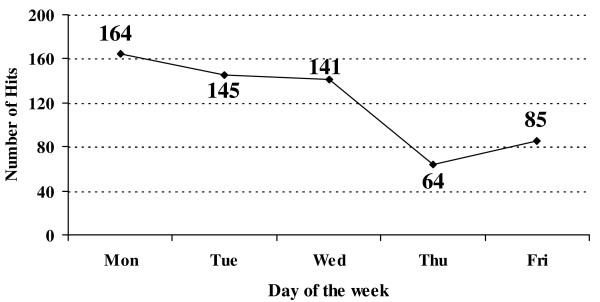
**Daily Access to NLM Server**. Number of hits to NLM server from Monday to Friday during the study period

**Figure 5:**
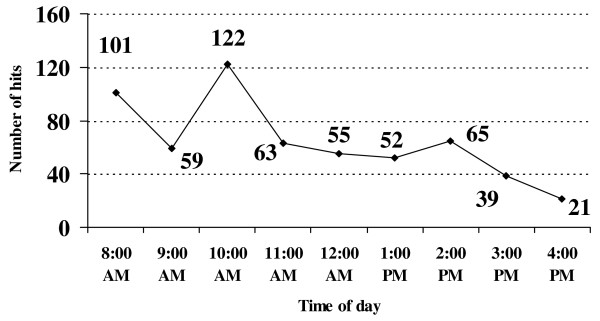
**Hourly Access to NLM Server**. Number of hits to NLM server hourly from 8 am to 4 pm during the study period

The analysis of NLM's Web server logs from August 2005 to February 2006 showed a cyclic pattern of usage, with peak usage during the months of December and January. On the other hand, the access dropped between September and November [Figure 3]. A total of 546 searches were performed using NLM tools during the seven-month period of study. Table 13 shows a monthly breakdown of specific NLM resources accessed by the participants. Eighty eight questions were sent to *ask*MEDLINE. The four most common questions were on cocaine and acute renal failure, tinnitus, hypernatremia, and arrhythmias in anemia. Two hundred and fifty five searches were carried out using PICO. The ten most frequently searched terms in PICO were colon cancer, mast cells, Crohn's disease, splenomegaly, pancytopenia, pancreatitis, systemic lupus, renal abscess, rhabdomyolysis and hypercalcemia. Disease Associations (DA) was used to perform two hundred and three searches. The ten more frequent searched associations were: pulmonary embolism and arthroscopy of the knee, stomatitis and recurrent herpes, asthma and magnesium, spirochetes in sputum, AIDS and Crohn's disease, vasculitis and purpura, adrenal insufficiency and eosinophilia, hepatomegaly and sarcoidosis, transudates and ovarian cancer, and kidney infarction and cocaine abuse. The weekly use of NLM tools showed a decreased use on Thursdays and Fridays from an initial three-day average of one hundred and fifty hits [Figure 4]. The time of major activity in the hospital wards correlated with the analysis of hourly access observed at the NLM server, which showed a maximum usage during the mornings, with a peak between 8 am and 10 am and progressive declining after 2 pm [Figure 5]. The devices were not available at night or weekends.

## Discussion

### Pre-study Internet and handhelds use survey

Our finding of 100% Internet use by physicians and 95% on a daily basis concurs with other surveys showing the trend towards a wide use of the Internet by clinicians in their daily practice [6,7]. The groups reported consistent use of the same set of resources but there were some differences between their preferences, both groups used in a similar proportion PubMed and UpToDate. However, the Interns group used more general and free resources to search for medical information on the Internet such as eMedicine, Google and Yahoo whereas the PostPGY1 group used more specific medical resources that required subscription such as MDConsult or NEJM. In addition, the difference in the utilization of resources was probably related to their education background, previous experiences, level of medical training and work requirements. There was a discrepancy between the higher numbers of Internet access reported in the survey and the data we obtained from the NLM server that could be explained by the participants' access to other non-monitored Websites as well.

Previous reports showed similar or higher rates of handhelds use among physicians than our survey [8,12,13]. However, we found an increase in usage from 17% to 44% between Interns and PostPGY1 groups. This is probably related to the increase of needs from work demands or from the influence of peers' and personal experiences as they progress through the residency training. Limited preparation on information technology and lack of confidence in using these devices usually prevent physicians from their use but after some time of exposure to the hybrid phone-PDAs they were more comfortable with them and almost three quarters of the participants considered obtaining one for their personal use. They were attracted by the smart phones' mobility and easy handling.

### Evaluation of smart phones usage

This study evaluated the feasibility of using smart phones as an alternative to access Web-based medical resources in a community-based internal medicine residency program. The setting of this group of physicians is common in the United States, a busy community hospital with no wireless network, few computers available for Internet access and the need for reliable and up-to-date medical information.

PDAs are useful clinical tools in improving preventive care and facilitating translation of knowledge into practice [14,15]. Our results indicate that about half of the participants (16/31) perceived a "frequent" global impact of their online searches in the diagnostic or management process and for 13 of them it happened "sometimes". Although this is a complex area to evaluate based on subjective judgments or without more objective information for analysis, a systematic review of observational studies suggested that the proportion of physicians who report such positive impact varies between 20% and 82% depending on the study, while nearly one-third of searches using information retrieval technology may have a positive impact on physicians [16]. However, further research is needed to obtain qualitative data to evaluate the impact on patient outcomes. The experience of new alternatives for clinical information retrieval at the point of care could be useful for many clinicians practicing in non-academic settings as well.

The daily usage of NLM tools showed an increased activity between 8 am and 2 pm. This pattern of utilization of the smart phones correlated with the time when medical teams were usually rounding in the wards, the purpose of this project. In the afternoons residents routinely attend to other outpatient activities and clinics, or leave the hospital if they are post-call. There is no clear explanation for the declining usage on Thursdays and Fridays. There were also evident variations in the monthly pattern of use of resources during the study period. The monthly change of team members is the most important factor considering that some of the residents were not comfortable using the devices or were not completely motivated to their use. The changes could be related also with the availability of the "champion" resident of the project in providing assistance and guidance to the ward teams, but not as a member of any of them. Another significant factor we considered influencing the results of this study is the lack of familiarity with this type of technology. Almost all the residents participating in the study were graduates of non-US medical schools, with no formal training in the use of handhelds and limited exposure to this equipment.

There were no reports of adverse effects or interference with medical devices in our study period while the smart phones were used in several hospital wards, emergency room, and critical care units. Although cellular telephones can interfere with medical equipment, a recent study showed a low incidence (1.2%) of clinically important interference. The devices currently in use are safer and must be close to medical equipment before any interference is noticed. The same study showed that the greatest distance at which interference occurred was 32 inches [17].

This study suffers from the methodological limitations common to single cohort study, including lack of randomization, a control group for comparison and qualitative data analysis that might help better understand the impact of medical information derived from the Internet by using smart phones. The results we obtained could have been biased due to the characteristics of the group, a small sample size as well as the level of education and training of the physicians before the study. The survey model used in the project provided subjective data and may introduce retrospective reported bias.

## Conclusion

In community hospitals and ambulatory clinics without wireless networks, where the majority of physicians work, real-time access to current medical literature is possible through smart phones. Proper training, technical support and familiarity with the technology will enhance the adoption of EBM practice. However, the presence of team leaders may be required until physicians recognize the value of information access at the point of care. New technologies for clinical information retrieval may benefit physicians' practice. In our study the real-time medical information obtained by the residents was perceived to be useful and impacted patients' care although additional studies in different settings are required to provide more objective data for an appropriate qualitative analysis in reference to the impact on patient management and outcome.

## Competing interests

The author(s) declare that they have no competing interests.

## Authors' contributions

SL and PF conceived of and developed the study and drafted the manuscript. LG assisted with drafting the manuscript and coordinating the study. MA assisted in writing the manuscript. FL assisted in the NLM's Web server logs data collection for analysis. All authors read and approved the final manuscript.

## Pre-publication history

The pre-publication history for this paper can be accessed here:



## Supplementary Material

Additional File 1 **Physicians' Internet and PDAs use survey (PDF)**. Questionnaire on Internet and handhelds usage Click here for file

Additional File 2 **Smart phones study final evaluation (PDF)**. Post-study feedback questionnaire on smart phones usage Click here for file

## Figures and Tables

**Table 1:** Pre-study survey. Proportion of the Internet access by location.

**Internet Access**	**PostPGY1 (n = 36) %**	**Interns (n = 24) %**
Home	45%	68%
Hospital – Office	55%	32%
Desktop	91%	85%
Mobile Computer	9%	15%

**Table 2:** Pre-study survey. Daily Internet usage.

**Daily Internet Usage**	**PostPGY1 (n = 36) n (%)**	**Interns (n = 21) n (%)**
<5 times/day	19 (53%)	12 (57%)
5–10 times/day	14 (39%)	7 (33%)
> 10 times/day	3 (8%)	2 (10%)

**Table 3:** Pre-study survey. Approximate number of hours on the Internet.

**Daily Internet Usage**	**PostPGY1 (n = 36) n (%)**	**Interns (n = 21) n (%)**
<1hours/day	3 (8%)	0
1–2 hours/day	23 (64%)	15 (71%)
>2 hours/day	10 (28%)	6 (29%)

**Table 4:** Pre-study survey. Reported proportion of the tasks done on the Internet.

**Internet Usage**	**PostPGY1 (n = 36) % time**	**Interns (n = 24) % time**
Personal information (includes e-mail)	22%	43%
Patient information (laboratory, test results)	30%	14%
General scientific information (medical knowledge)	23%	34%
Specific patient management questions	19%	9%
Others	6%	1%

**Table 5:** Pre-study survey. Websites used to search for general medical and scientific information.

**Website**	**PostPGY1 (n = 36) n (%)**	**Interns (n = 22) n (%)**
UpToDate	18 (50%)	9 (41%)
Google	18 (50%)	8 (36%)
MDConsult	18 (50%)	5 (23%)
PubMed	18 (50%)	5 (23%)
NEJM	17 (47%)	3 (13%)
eMedicine	14 (38%)	9 (41%)
Yahoo	0	6 (27%)

**Table 6:** Pre-study survey. Websites accessed for specific questions on patient management.

**Website**	**PostPGY1 (n = 33) n (%)**	**Interns (n = 18) n (%)**
UpToDate	18 (54%)	12 (66%)
Google	11 (33%)	1 (5%)
NEJM	10 (30%)	3 (16%)
PubMed	9 (27%)	5 (28%)
MDConsult	9 (27%)	6 (33%)
eMedicine	4 (12%)	3 (16%)

**Table 7:** Pre-study survey. Most common "EBM" resources cited.

**Website**	**PostPGY1 (n = 27) n (%)**	**Interns (n = 12) n (%)**
UpToDate	13 (48%)	5 (42%)
NEJM	13 (48%)	4 (33%)
PubMed	8 (30%)	5 (42%)
MDConsult	7 (26%)	5 (42%)

**Table 8:** Pre-study survey. Areas of interest on medical Internet training.

**Internet training Area of Interest**	**PostPGY1 (n = 35) n (%)**	**Interns (n = 24) n (%)**
Medical Knowledge	31 (89%)	22 (92%)
Clinical practice tools	28 (80%)	19 (79%)
EBM resources	31 (89%)	18 (75%)
Web pages design	11 (31%)	5 (21%)
Other	0	1 (4%)

**Table 9:** Pre-study PDA survey. Ownership and usage.

**PDA Usage**	**PostPGY1 (n = 36) n (%)**	**Interns (n = 24) n (%)**
PDA owners	16 (44%)	4 (17%)
Owned PDA > 1 year	10 (62%)	0
Owned PDA < 1 year	6 (38%)	4 (100%)
Training on PDAs use	none	none
Daily users	13 (81%)	2 (50%)
Using > 10 times/day	2 (12%)	1 (25%)
Use 5–10 times/day	8 (50%)	0
Use < 5 times/day	3 (19%)	1 (25%)

**Table 10:** Pre-study PDA survey. Reported uses of PDAs.

**PDA usage**	**PostPGY1 (n = 16) n (%)**	**Interns (n = 1) n (%)**
Pharmacopoeias	16 (100%)	1 (100%)
Medical books	14 (88%)	1 (100%)
Clinical tools	7 (44%)	1 (100%)
Calculator	7 (44%)	0
Address book	6 (38%)	0
Date book – Schedules	4 (25%)	1 (100%)
Text processing	1 (6%)	0
E-mail or Internet	1 (6%)	0
Patients' tracking	0	0

**Table 11:** Smart phones usage evaluation. How often the information was found on the Internet?

**Information found**	**PostPGY1 (n = 13) n (%)**	**Interns (n = 18) n (%)**
Always	0	3 (17%)
Frequently	6 (46%)	12 (66%)
Sometimes	7 (54%)	3 (17%)
Rarely	0	0
Never	0	0

**Table 12:** Smart phones usage evaluation. How often did the information have an impact in the diagnostic or management process?

**Perceived impact in diagnosis or management**	**PostPGY1 (n = 13) n (%)**	**Interns (n = 18) n (%)**
Always	0	0
Frequently	5 (38%)	11 (62%)
Sometimes	8 (62%)	5 (28%)
Rarely	0	1 (5%)
Never	0	1 (5%)

**Table 13:** Monthly access to NLM server by resource from August 2005 to February 2006.

**Month**	***ask*Medline n (%)**	**Disease Associations n (%)**	**PICO n (%)**	**Total (100%)**
August	21 (17%)	55 (45%)	46 (38%)	122
September	9 (23%)	5 (13%)	25 (64%)	39
October	8 (14%)	23 (40%)	27 (46%)	58
November	2 (18%)	1 (9%)	8 (73%)	11
December	15 (11%)	40 (31%)	76 (58%)	131
January	17 (11%)	69 (45%)	66 (44%)	152
February	16 (48%)	10 (30%)	7 (22%)	33
Total	88 (16%)	203 (37%)	255 (47%)	546

Values are number of searches logged at the NLM "PubMed for Handhelds" server only.
