# Supplementary material for: Evidence-based medicine among internal medicine residents in a community hospital program using smart phones
Source: BMC Med Inform Decis Mak. 2007 Feb 21;7:5. doi: 10.1186/1472-6947-7-5 (PMC1805745; doi:10.1186/1472-6947-7-5)
Supplement: Additional File 1 — Physicians' Internet and PDAs use survey (PDF). Questionnaire on Internet and handhelds usage [file 1472-6947-7-5-S1.pdf]

# PGHC PHYSICIANS' INTERNET AND PDA USE SURVEY: SECTION 1: INTERNET USE.

1. Do you use the Internet?
   a. YES ☐
   b. NO ☐…………..If NO, please go to the SECTION 2 (Question #13)

2. Where do you use Internet? Please estimate your usage.
   a. Home ________%
   b. Hospital or Office ________%
   c. Other ________%

3. How do you access the Internet? Please estimate from where you access it.
   a. Desktop ________%
   b. Mobile computer________%
   c. Handheld device ________%
   d. Phone/PDA hybrid ______%

4. How often do you access the Internet? Please estimate the number of times
   a. Daily _______times
   b. Weekly _______times
   c. Monthly _______times

5. How many hours do you spend surfing the Web?
   a. Daily ______hrs
   b. Weekly ______hrs
   c. Monthly ______hrs

6. Please estimate the distribution of the time spend on the Internet :
   a. Personal information _________% (includes e-mail)
   b. Patient's clinical information _________% (laboratory, tests results)
   c. General scientific information _________% (Medical knowledge)
   d. Specific patient management _________% (Individual questions)
   e. Others _________%

7. What are your favorite Web sites for GENERAL medical, scientific information:
   a. ____________________________
   b. ____________________________
   c. ____________________________
   d. ____________________________
   e. ____________________________

8. Mention the Web sites you use for SPECIFIC questions related to patient management:
   a. ____________________________
   b. ____________________________
   c. ____________________________
   d. ____________________________
   e. ____________________________

9. Mention the Evidence-Based Medicine resources you know or use.
    a. ______________________________
    b. ______________________________
    c. ______________________________
    d. ______________________________
    e. ______________________________

10. Would you like training on the use of Internet for medical applications?
    a. YES  ☐ …..If Yes, please go to question # 11
    b. NO  ☐ …...If No, explain reason(s)…………………………………….................

11. If YES, please specify your level of interest:
    ☐5 I prefer it now
    ☐4 As soon as possible
    ☐3 Within six months
    ☐2 Sometime during the year
    ☐1 Next year

12. Please check your specific area(s) of interest
    a. Medical knowledge resources
    b. Tools for clinical practice
    c. Evidence-Based Medicine resources
    d. Web pages design
    e. Other applications:__________________________________________________

**PHYSICIANS' INTERNET AND PDA USE SURVEY:**
**SECTION 2 – PDAs Use.**

13. Do you own a personal digital assistant (PDA)?
    a. YES ☐
    b. NO ☐………………if NO, please answer Question # 14 only

14. If NO, are you planning to buy one in the near future?
    a. YES ☐
    b. NO ☐
    c. Don't Know ☐

15. What type of PDA do you use?
    a. ___________________________Model____________________

16. Is your PDA wireless enabled? (Bluetooth, Wi-Fi, etc)
    a. YES ☐
    b. NO ☐

17. How long have you had your PDA?
    a. _________Weeks
    b. _________Months
    c. _________Years

18. How did you learn to use your PDA? (Check all that apply)
    a. ☐ Self Taught
    b. ☐ Peers
    c. ☐ PDA manual
    d. ☐ Institutional training
    e. ☐ Internet guides
    f. ☐ Others_______________________
    g. ☐ I don't know how to use it yet

19. How often do you use your PDA?
    a. ☐ #_______ times per day
    b. ☐ #_______ times per week
    c. ☐ #_______ times per month
    d. ☐ I rarely use it
    e. ☐ It's still in the box

20. Please estimate how you use your PDA:
(Please include the name of the software, applications on the right side)

a. ☐ Date book – Schedules _________% 
b. ☐ Address book _________% 
c. ☐ Calculator _________% 
d. ☐ Pharmacopeias _________% ______________________________________
e. ☐ Medical references _________% ______________________________________
f. ☐ Text processing _________% ______________________________________
g. ☐ Clinical tools _________% ______________________________________
h. ☐ Patients' tracking _________% ______________________________________
i. ☐ E- mail or Internet _________% ______________________________________
j. ☐ Other programs _________% ______________________________________

21. Would you like training on the medical uses of PDAs?

a. YES ☐...…. If Yes, please go to question # 22
b. NO ☐…… If No, please explain reason(s)………………………………………….

22. If YES, please specify your level of interest:

☐5 I prefer it now
☐4 As soon as possible
☐3 Within six months
☐2 Sometime during the year
☐1 Next year

NAME: ……………………...……………………………………..…..

E-Mail: ………………………………………………………………..
