# Supplementary material for: Evidence-based medicine among internal medicine residents in a community hospital program using smart phones
Source: BMC Med Inform Decis Mak. 2007 Feb 21;7:5. doi: 10.1186/1472-6947-7-5 (PMC1805745; doi:10.1186/1472-6947-7-5)
Supplement: Additional File 2 — Smart phones study final evaluation (PDF). Post-study feedback questionnaire on smart phones usage [file 1472-6947-7-5-S2.pdf]

PGHC RESIDENCY PROGRAM
WIRELESS INTERNET ACCESS PROJECT
FINAL EVALUATION:
Name: ______________________________Date___________

1. Did your team use the PDA for wireless Internet access during the daily wards activities?
    a. YES ☐
    b. NO ☐

2. Was this your first experience with this type of Internet access to look for medical information?
    a. YES ☐
    b. NO ☐

3. How many times a day did your team use the device? (on average)
   ☐ 1 to 5
   ☐ 5 to 10
   ☐ More than 10

4. Please rate the Internet connection?
   ☐5 Very Fast
   ☐4 Fast
   ☐3 Average
   ☐2 Slow
   ☐1 Very slow

5. Is the PDA easy to use?
   ☐5 Very easy
   ☐4 Easy
   ☐3 Fair
   ☐2 Difficult
   ☐1 Very difficult

6. Mention the main Web sites or Web-based tools you used to get medical information:

   ____
   ____
   ____
   ____
   ____
   ____

7. How often did you **find** the information you were looking for?
   ☐5 Always
   ☐4 Frequently
   ☐3 Sometimes
   ☐2 Rarely
   ☐1 Never

8. The information obtained had **impact** in the diagnostic or management process:
   - 5 Always
   - 4 Frequently
   - 3 Sometimes
   - 2 Rarely
   - 1 Never

9. How likely are you to use these medical applications in the future?
   - 5 Very likely
   - 4 Likely
   - 3 Not sure
   - 2 Not likely
   - 1 Not at all likely... explain reason(s)…………………………………....

10. Are you planning to buy a PDA with wireless internet access for personal use?
    a. YES b. NO
    If YES, please specify your level of interest:
    - 4 As soon as possible
    - 3 Soon (less than 6 months)
    - 2 In the near future (6 to 12 months)
    - 1 Later

11. Overall, how satisfied are you with this experience?
    - 4 Very satisfied
    - 3 Satisfied
    - 2 Neither satisfied nor dissatisfied
    - 1 Very dissatisfied

12. Would you recommend these devices to colleagues for their daily use?
    a. YES ……..Why?..
    b. NO …….Why?...

13. Please indicate the ADVANTAGES of this type of PDAs use:
    ____
    ____
    ____

14. Please indicate the DISADVANTAGES or BARRIERS for the use of these devices:
    ____
    ____
    ____

15. The information provided for the devices and Web tools used was:
    - 5 Clear
    - 4 Easy to follow
    - 3 Enough
    - 2 Unclear
    - 1 Not enough

16. Do you have any additional recommendations or comments about this project?
    ____
    ____
